# Slow recovery rates and spatial aggregation of *Triatoma infestans* populations in an area with high pyrethroid resistance in the Argentine Chaco

**DOI:** 10.1186/s13071-024-06366-7

**Published:** 2024-07-02

**Authors:** María Carla Cecere, María Sol Gaspe, Natalia Paula Macchiaverna, Gustavo Fabián Enriquez, Alejandra Alvedro, Mariano Alberto Laiño, Julián Antonio Alvarado-Otegui, Marta Victoria Cardinal, Ricardo Esteban Gürtler

**Affiliations:** 1 Departamento de Ecología, Genética y Evolución, Facultad de Ciencias Exactas y Naturales, Universidad de Buenos Aires, Buenos Aires, Argentina; 2 Instituto de Ecología, Genética y Evolución de Buenos Aires (IEGEBA), Consejo Nacional de Investigaciones Científicas y Técnicas (CONICET), Universidad de Buenos Aires, Ciudad Universitaria, C1428EGA Buenos Aires, Argentina; 3 Instituto de Zoonosis Luis Pasteur, Av. Díaz Vélez, 4821 Buenos Aires, Argentina

**Keywords:** Gran Chaco, Vector control, Hotspots, Pyrethroid resistance, *Triatomainfestans*, Spatial heterogeneity, Disease elimination

## Abstract

**Background:**

The emergence of pyrethroid resistance has threatened the elimination of *Triatoma infestans* from the Gran Chaco ecoregion. We investigated the status and spatial distribution of house infestation with *T. infestans* and its main determinants in Castelli, a municipality of the Argentine Chaco with record levels of triatomine pyrethroid resistance, persistent infestation over 2005–2014, and limited or no control actions over 2015–2020.

**Methods:**

We conducted a 2-year longitudinal survey to assess triatomine infestation by timed manual searches in a well-defined rural section of Castelli including 14 villages and 234 inhabited houses in 2018 (baseline) and 2020, collected housing and sociodemographic data by on-site inspection and a tailored questionnaire, and synthetized these data into three indices generated by multiple correspondence analysis.

**Results:**

The overall prevalence of house infestation in 2018 (33.8%) and 2020 (31.6%) virtually matched the historical estimates for the period 2005–2014 (33.7%) under recurrent pyrethroid sprays. While mean peridomestic infestation remained the same (26.4–26.7%) between 2018 and 2020, domestic infestation slightly decreased from 12.2 to 8.3%. Key triatomine habitats were storerooms, domiciles, kitchens, and structures occupied by chickens. Local spatial analysis showed significant aggregation of infestation and bug abundance in five villages, four of which had very high pyrethroid resistance approximately over 2010–2013, suggesting persistent infestations over space-time. House bug abundance within the hotspots consistently exceeded the estimates recorded in other villages. Multiple regression analysis revealed that the presence and relative abundance of *T. infestans* in domiciles were strongly and negatively associated with indices for household preventive practices (pesticide use) and housing quality. Questionnaire-derived information showed extensive use of pyrethroids associated with livestock raising and concomitant spillover treatment of dogs and (peri) domestic premises.

**Conclusions:**

*Triatoma infestans* populations in an area with high pyrethroid resistance showed slow recovery and propagation rates despite limited or marginal control actions over a 5-year period. Consistent with these patterns, independent experiments confirmed the lower fitness of pyrethroid-resistant triatomines in Castelli compared with susceptible conspecifics. Targeting hotspots and pyrethroid-resistant foci with appropriate house modification measures and judicious application of alternative insecticides with adequate toxicity profiles are needed to suppress resistant triatomine populations and prevent their eventual regional spread.

**Graphical Abstract:**

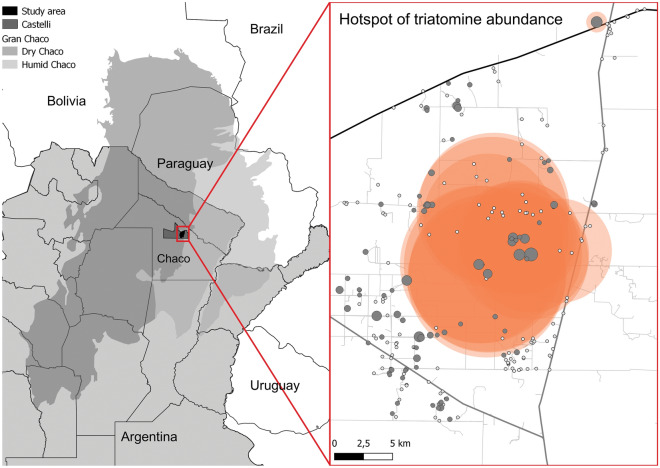

**Supplementary Information:**

The online version contains supplementary material available at 10.1186/s13071-024-06366-7.

## Background

Chagas disease, caused by the protozoan *Trypanosoma cruzi*, is the most important neglected tropical disease in Latin America and a source of growing concern in non-endemic countries through international migration [1]. In endemic areas, *T. cruzi* is mainly transmitted by triatomine bug species that establish domestic colonies. *Triatoma infestans* (Klug 1834) (Hemiptera: Reduviidae) has historically been the main vector of *T.* *cruzi* in South America. The core of the distribution range of *T.* *infestans* is in the Gran Chaco ecoregion, the second largest forest biome after the Amazon. These forests, extending over 23 million ha across the Argentine Chaco region, have experienced rapid land use change [2]. Insecticide-based control programs of major triatomine species have achieved variable degrees of success, including those targeting *T.* *infestans* in the Southern Cone countries of South America [3, 4]. House spraying with pyrethroid insecticides has usually been much more effective in domestic than in peridomestic habitats where the insecticides meet harsh environmental conditions [5, 6].

The domestic transmission of *T.* *cruzi* is shaped by ecological, biological, and social factors that jointly determine habitat suitability for domestic triatomine species [4]. These factors directly affected the vital rates and abundance of *T.* *infestans* [7–10], *Triatoma pallidipennis* [11], and *Triatoma dimidiata* [12, 13], among others. The type of materials and degree of repair of walls and roofs determine housing quality and the amount of suitable refuges for *T*. *infestans*. Domestic infestation and bug abundance were associated positively with host availability (e.g. indoor-nesting chickens, overcrowding, number of human residents) and negatively with the domestic use of insecticides by householders [10, 14, 15].

House infestation after insecticide spraying has often been associated with the occurrence of local residual foci, external triatomine sources (including sylvatic foci), technical glitches, and reduced persistence of insecticides depending on substrate features [5, 16–21]. The emergence of pyrethroid resistance in *T. infestans* across large sections of the Gran Chaco ecoregion [22] and in other triatomine species [23, 24] added a new source of concern. The occurrence of pyrethroid-resistant *T. infestans* was related to three measures of temperature and two of rainfall [25].

Severe control failures of *T. infestans* in sections of northern Argentina over the late 1990s [26] were linked to high levels pyrethroid resistance, an unexpected phenomenon later detected across the Andean valleys in Bolivia [22]. Maximum resistance levels to pyrethroids (deltamethrin) were also detected in several rural villages of Castelli municipality (Chaco Province) in northeastern Argentina around 2010–2013 [27–30] and in Salta, Tarija, and Cochabamba [31]. The high levels of pyrethroid resistance in Argentine and Bolivian foci of *T. infestans* have been linked mainly to two nucleotide mutations (L1014 and L925I) in the sodium channel gene (*kdr*, knockdown resistance) [29]. Other mechanisms involved enhanced detoxification by oxidases (metabolic resistance) and the amount of surface hydrocarbons and cuticle thickness, which reduce the penetration of insecticide molecules [32]. The L925I mutation and the other mechanisms mentioned above also occurred in pyrethroid-resistant *T. infestans* from Castelli [33–35], which were susceptible to fenitrothion [36]. As federal health authorities banned the indoor spraying with organophosphate and carbamate insecticides, in the absence of registered alternatives, triatomine control programs gradually discontinued or stopped pyrethroid applications in villages with evidence of high pyrethroid resistance. The subsequent fate of resistant foci in the absence of further spraying with pyrethroids remained undetermined by 2018 despite the risk of transmission they presumably posed and the potential expansion of pyrethroid-resistant *T. infestans* across the region; neither was the spatiotemporal distribution of *T. infestans* populations in this district investigated or its ecological and sociodemographic determinants.

The pyrethroid-resistant foci in Castelli were the likely immediate sources of more limited foci of pyrethroid-resistant *T. infestans* in the neighboring Pampa del Indio municipality over 2008–2009 [18]. Sustained surveillance-and-response interventions over a decade nearly suppressed house infestation and triatomine infection in Pampa del Indio [21, 37], and transmission of *T. cruzi* to humans was interrupted by 2017 [38]. Follow-up surveys of Pampa del Indio houses lying on the border with Castelli municipality detected new foci of *T. infestans* over 2016–2017 which tested pyrethroid resistant (Gaspe et al. unpublished results). These findings motivated the search for its possible sources in neighboring Castelli and for alternative control tools. A field trial of fluralaner administered to dogs was launched over 2018–2019 [39], and interventions were scaled up in February 2020 with the goals of reducing or suppressing house infestation and transmission risk.

As part of this broader endeavor, we assessed house infestation and the relative abundance of *T. infestans* by type of habitat across 14 rural villages in a well-defined area of Castelli in both 2018 and 2020 and mapped the spatial distribution of houses and infestations. We anticipated higher levels of house infestation and triatomine abundance in villages that in the past had shown high pyrethroid resistance, with concomitant clustering patterns, compared to neighboring districts with little or no pyrethroid resistance. We conducted a risk factor analysis of the main ecological and sociodemographic determinants associated with baseline house and domestic infestation and abundance. Because pyrethroid-resistant *T. infestans* differ from susceptible conspecifics in multiple biological traits (e.g. [40, 41]), we examined whether the eco-bio-social determinants of house and domestic infestation identified elsewhere operated in Castelli, with special attention to household pesticide use. Based on consistent evidence across the Argentine Chaco [6, 9, 42, 43], we expected that domiciles, kitchens, storerooms, and structures used by chickens would harbor heavier infestations. An important premise of this research is that a better understanding of the location, scale, and driving factors of house infestation and triatomine abundance may enhance the chances of developing and implementing appropriate control actions by targeting key areas, villages, and bug habitats instead of investing the limited resources in traditional blanket interventions. This approach is even more pertinent in areas with high pyrethroid resistance.

## Methods

### Study area

**Fig. 1:**
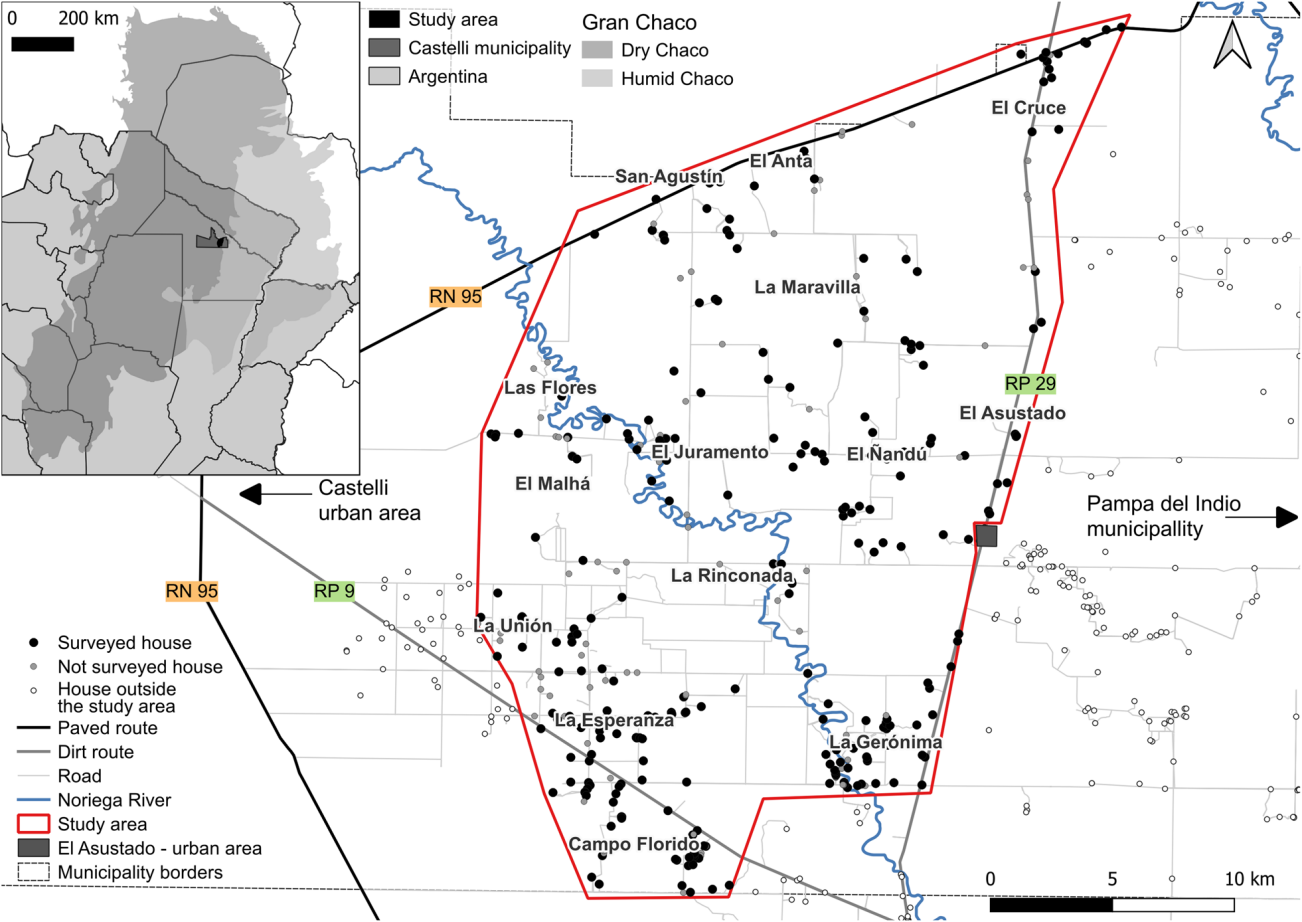
Map of the study area showing the surveyed houses (dots) and villages in castelli municipality. Inset shows the study area within Güemes Department, Chaco Province (Argentina), and the Gran Chaco region

Fieldwork was carried out in a well-defined rural section of the municipality of Juan José Castelli (25°57′00′S, 60°37′00′W), General Güemes Department, Chaco Province (Fig. 1). Güemes Department historically ranked at the top of districts in Chaco in terms of the seroprevalence of human infection with *T. cruzi* [44] and vulnerable living conditions affecting both indigenous and creole groups. Following a ruling of Argentina’s Supreme Court of Justice in 2007 for prioritized healthcare interventions and blocking vector-borne transmission of *T. cruzi* across Güemes Department, Chagas vector control programs from several provinces intensified coordinated control efforts throughout Güemes and repeatedly assessed house infestation by timed manual searches and sprayed house premises with insecticides over 2007–2014. Spray coverage and the frequency of treatments varied widely over time and space. Extensive land use changes occurred over this period, as across the Gran Chaco between 2000 and 2021, where changes involved a large increase of the area for agriculture and livestock farming at the expense of natural woody vegetation (http://chaco.mapbiomas.org, accessed 5 March 2024). The study area (delimited by a red line in Fig. 1 and including a buffer zone extending up to 1.2 km from Provincial Route 29, i.e. eastern boundary) encompassed 14 villages regrouped in 11 groups based on house proximity: La Maravilla and five nearby houses from San Agustín and El (or La) Anta; El Asustado; El Malhá (or Malá) and one nearby house from Las Flores; La Rinconada; La Unión; La Gerónima; Campo Florido; El Cruce; El Juramento; El Ñandú and La Esperanza (Fig. 1). While adjacent villages from Pampa del Indio on the eastern border with Castelli lacked foci of *T. infestans* [21], rural settlements along other borders were infested. The villages were in a mosaic landscape of fields destined to agriculture and native xerophytic forest showing different degrees of degradation [45].

Official records showed that the study villages were treated with pyrethroid insecticides until 2014, with no further records found thereafter. A small control trial of house infestation with triatomines based on treatment of dogs with fluralaner encompassed seven rural houses from El Ñandú and El Asustado in March–April 2018 [39]. Dose-mortality data of deltamethrin applied to first-instar nymphs of *T. infestans* collected around 2010–2013 indicated that resistance ratios [RR_50_, calculated by dividing the median lethal dose (LD50) of a tested field population by the LD50 of the susceptible or reference population] ranged from 233 to 2000 at El Juramento, El Malhá, El Asustado, La Rinconada, El Ñandú, and La Esperanza and was 3.9 at La Gerónima [31]. Pyrethroid resistance levels were classified as high (RR_50_ > 100), medium (10 < RR_50_ < 100) and low (RR_50_ < 10) [31].

### Site-level infestation and ecotope

The local population was of Creole descent. House compounds comprised human sleeping quarters (i.e. domicile) and separate peridomestic structures (i.e. peridomicile); the latter included storerooms, kitchens, mud ovens, latrines, sites occupied by chickens or other poultry (trees, coops, nests, nesting structures), and corrals for goats or sheep, pigs, cattle, and horses. Each habitat type was considered an ecotope. House compounds sometimes had more than one separate domicile used as sleeping quarters by relatives. Chicken nesting structures (‘nideros’) usually comprised an elevated platform made of wood or bricks where chickens (occasionally ducks or turkeys) brooded or roosted [9]. Poultry, pigs, and goats were raised for subsistence. A site is any individual structure (built or given a defined use by householders) that provides shelter and a bloodmeal source to triatomines.

### Study design

We conducted a 2-year longitudinal survey of house infestation in 14 rural villages inspected in October–November 2018 (baseline) (Fig. 1). A follow-up survey, comprising the cohort of inhabited houses at baseline, used the same methods in February 2020.

### Village- and house-level infestation

At baseline, we showed householders dry specimens of *T. infestans*, *Triatoma sordida*, and other morphologically similar insects and asked for the presence of triatomines within their premises. All existing house compounds and public buildings were identified with a numbered plaque and georeferenced using a global positioning system (Garmin Legend). All sites within each house compound were searched for triatomines by timed manual collections (TMC) conducted by skilled technicians affiliated to government-sponsored Chagas vector control programs (National and Provincial). Searches used 0.2% tetramethrin spray (Espacial, Argentina) as a dislodging agent and a fixed capture effort (one person during 15 min per site). All triatomines were identified taxonomically and counted per site according to species, stage, and sex at the field laboratory as described [46]. A house was considered “infested” or “positive” if any live *T. infestans* (barring eggs) was found by TMC. “Colonized” or “colonization” meant the finding of at least one live nymph of *T. infestans*. The relative abundance (total catch) of triatomine bugs was calculated as the number of live bugs collected by TMC per unit effort at defined spatial scales (i.e. house, domicile, peridomicile, and site).

### Housing characteristics, sociodemographic variables, and house infestation

We interviewed householders using a modified questionnaire [15] to record the names, age, and education level of each resident; age of the house; the household numbers of dogs, cats, chickens (fowl), and corralled animals (goats, pigs, goats or sheep, cattle, and horses), and the typical structures used by dogs, cats, and chickens for resting, with special consideration of their numbers indoors. We also inspected the premises to record several housing characteristics: number and area of sleeping quarters, the building materials used in the roofs, walls and floors of domiciles, the number of separate domiciles and peridomestic structures by main function (storeroom, kitchen, various types of structures used by chickens, corral, bathroom, and others), and a sketch map of their location. Domestic refuge availability was assessed visually by trained team members and categorized from 1 (no refuge) to 5 (maximum refuge) based on the type of construction materials, condition of wall plaster, type of plaster (cement, mud, or none), and the presence of cracks and crevices where triatomines could hide [9]. The collected data were used to compute the goat-equivalent index (which quantifies the household number of cattle, pigs, goats, and poultry owned by each household, expressed in terms of goat biomass [15]) and a residential overcrowding index (defined as the number of human residents per sleeping quarter), with three or more occupants taken as critical overcrowding by the federal government census bureau. Household educational level is the mean number of schooling years attained by household members aged ≥ 15 years old.

We recorded the use of insecticides by householders [with fixed options for aerosols, creoline, Sevin (a carbamate, Sevin^®^, Bayer), deltamethrin or cypermethrin, fumigant canisters and others], with each type scored for frequency, site of application, and whether backpack manual compression sprayers were used. We also collected household-level data on the type and frequency of application of antiparasitic products to dogs, cats, and farm animals and the date of the last house spray with insecticide conducted by government-sponsored vector control personnel and their geographic provenance.

### Longitudinal survey of infestation and triatomine abundance

Both the baseline and follow-up surveys classified each existing house unit as occupied (i.e. inhabited, regardless of whether the residents were temporarily absent); closed (inhabited with no resident available during one or two visits); vacant (no sign of occupancy, often corroborated by neighbors); demolished: no longer existing at its prior location; and new (i.e. a newly built housing unit not previously recorded at its current location).

### Data analysis

We restricted the analysis of baseline infestation with *T. infestans* to 234 inhabited rural houses inspected by TMC in October–November 2018; these included 12 houses with only domestic habitats inspected, two houses with only peridomestic habitats inspected, and seven houses participating in a small trial of fluralaner administered to dogs in March–April 2018 [39]. For seven fluralaner-treated houses only, we used the preintervention (2018) estimates of triatomine infestation and abundance, and excluded these houses from the 2020 data set. We excluded from the 2018 analysis of infestation 90 houses that could not be accessed for inspection (46 were vacant, 42 closed, and 2 households refused to participate) and 27 public buildings unlikely to be infested.

The prevalence of infestation and colonization with *T. infestans* was calculated at both the site and house levels. Agresti-Coull binomial 95% confidence intervals (95% CI) were used for infestation [47]. Missing data for some variables as of 2018 (i.e. age of house, time since last reported insecticide spraying, site of insecticide application) were completed using data collected in February 2020.

Using multiple correspondence analysis (MCA), we constructed three indices to synthesize the multiple dimensions of housing quality (various building characteristics restricted to domiciles), domestic host availability (based on the household number of persons, dogs, cats, and chickens, and the presence of poultry indoors), and household preventive practices (based on the reported use of each type of pesticide) at baseline. The housing quality index reflected the combination of building materials of walls (mud walls, brick-cement walls, and a mixture of both categories) and roofs (corrugated metal-sheets, other materials such as thatched roofs, and a mixture of both types), the degree of wall cracking (none, few, and many cracks), and condition of wall plaster (full, partial, none). The domestic host availability index summarized the number of potential hosts of *T. infestans* (adult and child residents, household number of dogs, cats, and chickens, and the presence of chickens nesting indoors) [48]. The index for household preventive practices combined the reported use of any pesticide in domiciles or peridomiciles and of each type of pesticide (categorized as aerosol sprays, phenolic disinfectants or acaricides, pyrethroids, carbamates, mosquito coils, and other types). Information on livestock treatment frequently had missing data and therefore was not included in this index. For MCA, both continuous and discrete variables were categorized according to their quartiles. This analysis reduces the dimensionality of the covariance matrix in linear combinations of the original variables [49]. MCA biplots (with dimension 1 on the horizontal axis and dimension 2 on the vertical axis) describe graphically the pattern of relationships among the different categorical variables used to build the three indices. To examine the associations among categories, we plotted the first two dimensions of the Euclidean space. As the first dimension (inertia) accounts for most of the variance, the score for each household was used as a quantitative index [50]. For a better interpretation, the indices were considered as dimension 1.

We first performed bivariate logistic and negative binomial regression of house infestation and bug abundance at baseline (response variables) on each selected explanatory variable with supporting evidence [9, 15]. These analyses offer insight into unadjusted associations and allow comparisons with the outcomes from other studies including our own ones. We also focused on domestic infestation, as it is strongly connected with the risk of human infection, and house-level infestation, the usual metric used by triatomine control programs. We then tested the relationship between house or domestic infestation (binary response variables) and selected explanatory variables using multiple logistic regression. The same explanatory variables were analyzed for relative bug abundance using negative binomial regression. The models included five or six explanatory variables: goat equivalent index; indices for domestic host availability, household preventive practices and housing quality; distance to the nearest infested house and the relative abundance of *T. infestans* in peridomestic habitats (restricted to models of domestic infestation and domestic bug abundance). The total number of sites inspected by TMC at the house or domicile levels was added as an offset term. All continuous explanatory variables (household preventive practices, housing quality, domestic host availability, goat-equivalent index, and distance to the nearest infested house) were standardized to compare the scores measured in different scales. No multicollinearity issue was detected by variance inflation factors and other diagnostics implemented in Stata 15.1 [51].

### Spatial patterns

The entomological database was associated with the geographic coordinates of each house (in Universal Transverse Mercator, Zone 20S, WGS1984 datum). We calculated the matrix of distances to the nearest infested house using QGIS 3.4 [52]. Global spatial analyses determined the occurrence of clustering anywhere within the study area via the K-function (for house infestation) and the weighted K-function (for bug abundance) using random labeling as the null hypothesis [53, 54]. “Cluster” refers to an unusual aggregation of locations with high triatomine abundance or infested houses in time and space. For each analysis, we used L (linearized K-function) and L_w_ (linearized weighted K-function) statistics against the point pattern locations of all houses. We performed 25 cycles of 999 Monte Carlo simulations and calculated the 95% confidence envelope with the 25th upper and lower simulations for each cycle. A local spatial statistic, Getis Gi*(d), was used to identify the precise location of clusters or “hotspots” by comparing the values at all locations j within specified distances (*d*) of the location under consideration. We then mapped the occurrence of clusters with high bug abundance at the house level. This analysis distinguishes between positive and negative aggregations of events. Clustering was evaluated up to a radius of 6.5 km (i.e. one-third of the shortest dimension of the polygon) in 100-m radius increments, and parameter settings were the same as for global and local analysis. Spatial analyses use ‘spatstat’ and ‘spdep’ package in R [55]. Other statistical analyses used Stata 15.1 [56].

## Results

### Longitudinal survey of infestation and triatomine abundance

The overall prevalence of house infestation with *T. infestans* in the closed cohort of houses inspected in both 2018 (33.8%) and 2020 (31.6%) virtually matched the historical grand average for the intervention period 2005–2014 (33.7%) (Table 1). Median bug abundance varied little between six and eight triatomines per infested house over 2018–2020. While peridomestic infestation remained the same (26.4–26.7%) between 2018 and 2020, domestic infestation decreased slightly from 12.2 to 8.3%, respectively. Domestic and peridomestic bug abundance remained stable over 2018–2020.

**Table 1:** Infestation, median abundance and total catch of *Triatoma infestans* in houses, domiciles, and peridomiciles inspected in 2018 and 2020 in Castelli, including data reported in [57] for the intervention period 2005–2015

Habitat level	% Infestation (no. of units inspected)			Median bug abundance (Q1–Q3, total catch)	
	2005–2015^a^	2018	2020^b^	2018	2020^b^
House	33.7(3342)	33.8 (234)	31.6 (193)	8 (3–15; 888)	6 (2–14; 566)
Domicile	24.3	12.2 (222)	8.3 (193)	4 (1–9; 131)	4 (2–4; 69)
Peridomicile	16.8	26.7 (232)	26.4 (193)	8 (3–16; 757)	6 (2–16; 497)

^a^Taken from Table 3 [57] by summing up the year-specific numbers of evaluated and infested observation units at the house, domestic, and peridomestic levels over 2005–2014; total numbers recalculated from row data

^b^Seven fluralaner-treated houses were excluded

In total, 41 houses from the 2018 cohort were lost to follow-up in 2020, including houses that were closed (*n* = 19), vacant (*n* = 12), refused to participate in the triatomine survey (*n* = 2), and demolished (*n* = 1) and had been treated with fluralaner (*n* = 7) in the interim. Among the 193 houses inspected for infestation in both 2018 and 2020, 49 (25.3%) were observed positive for *T. infestans* in both surveys (co-positive); 115 (59.6%) were observed negative in both surveys (co-negative); 12 (6.2%) initially negative houses were subsequently observed positive, and 17 (8.8%) initially positive houses were subsequently negative (McNemar’s χ^2^ = 0.86, *df* = 1, *P* = 0.35). Positive-to-negative transitions included low-density infestations (median, 3 bugs per unit effort; Q1–Q3: 1–4; range: 1–33; *n* = 17), and so did negative-to-positive transitions (median, 3; Q1–Q3: 1–7; range: 1–17; *n* = 12). In domiciles, 10 houses were co-positive, 157 were co-negative, 5 converted from negative to positive, and 11 reversed from positive to negative (McNemar’s χ^2^ = 2.25, *df* = 1, *P* = 0.13). Householders from 13 (6.7%) of the 193 houses inspected reported that their premises were treated with insecticide using backpack manual sprayers between 2018 and 2020.

### Village- and house-level infestation

Baseline domestic infestation varied from 0 to 31.3% across villages (Table S1). Villages where house infestation was < 30% had no detectable domestic infestation. Most of the study houses (59.8%) pertained to the seven villages that had displayed RR_50_ > 3 [57]; 73.6% of the houses within this group belonged to villages with RR_50_ > 100 (Table S1). In total, we collected 888 *T. infestans* in 112 (6.4% of 1746) sites inspected by TMC at baseline (including 31 of 256 domestic sites and 81 of 1490 peridomestic sites); 85.2% of all triatomines were caught in peridomiciles. The stages most frequently captured were fifth-instar nymphs (32%), males (24%), and females (14%).

**Fig. 2:**
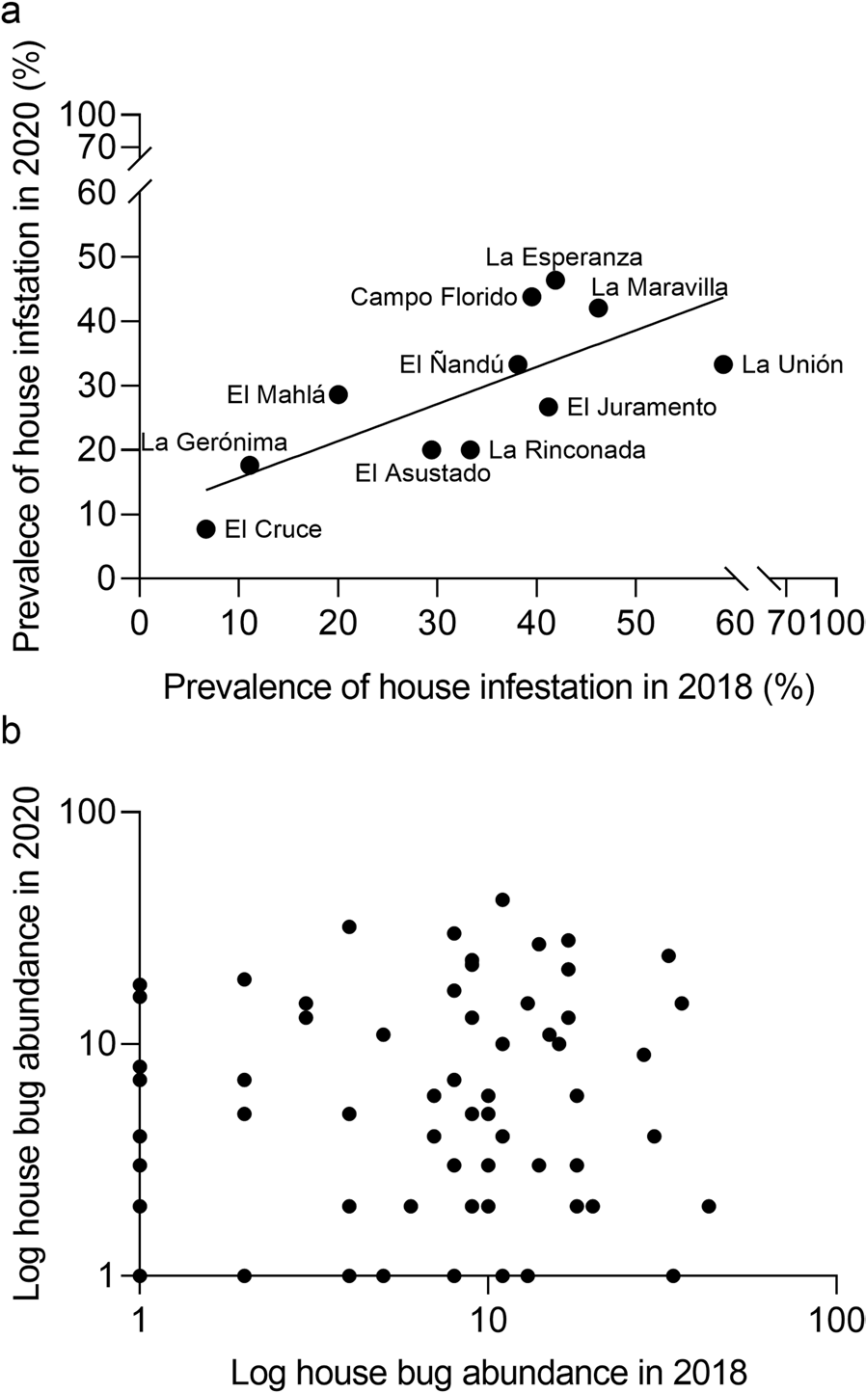
Village-level house infestation with *Triatoma infestans* (**a**) and median bug abundance (**b**) in 2018 and 2020 in Castelli

Village-level estimates of the proportion of houses infested with *T. infestans* (including 234 houses in 2018 and 193 houses in 2020) correlated positively and significantly over time (Fig. 2a; ordinary linear regression: *y* = 0.5742 *x* + 0.0993; *n* = 11; adj *R*^*2*^ = 0.485, *P* = 0.01). Log-transformed bug abundance (+ 1) at the house level in 2020 and 2018 was highly significantly correlated (*y* = 0.6238 *x* + 0.0787, *n* = 193; adj *R*^*2*^ = 0.423, *P* < 0.001) (Fig. 2b).

### Site-level infestation and ecotope

**Fig. 3:**
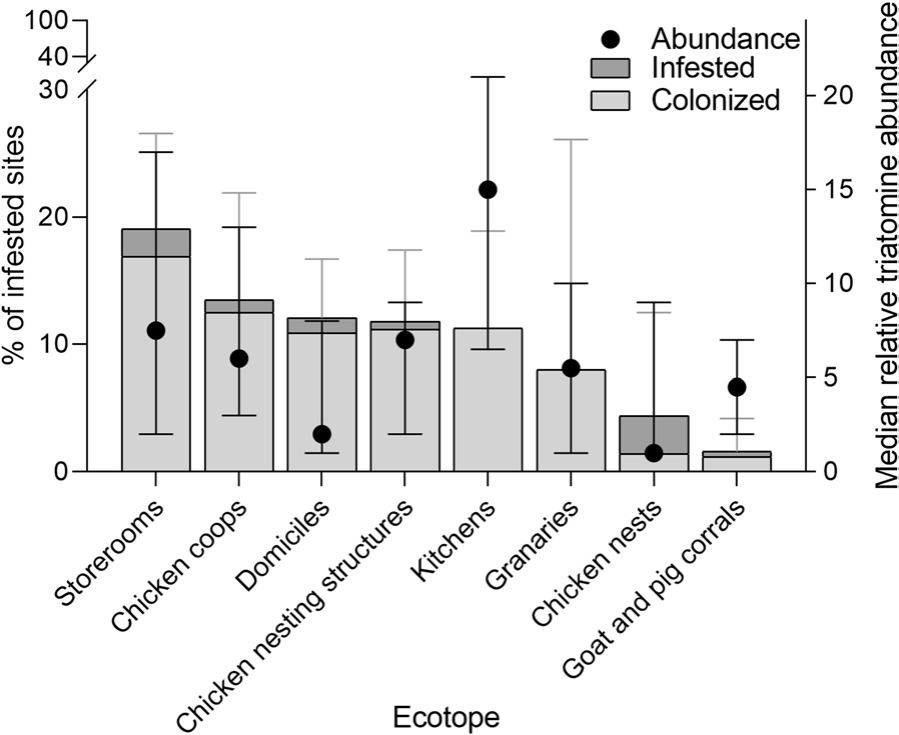
Prevalence of site-level infestation and colonization with *Triatoma infestans* (bars) and median relative bug abundance per house (dots) according to the type of ecotope with at least one infested site in Castelli at baseline, 2018. Whiskers indicate the upper and lower limits of Agresti-Coull 95% confidence intervals for mean infestation (gray bars) and the first and third quartiles (dark bars) of median bug abundance

*Triatoma infestans* occupied domiciles and a wide range of peridomestic ecotopes at baseline (Fig. 3). No *T. infestans* was caught in cow or horse corrals, trees or sticks with chickens, bathrooms (latrines), piled materials, sheds, mud ovens, and poultry scaffoldings (“cimbras”). Ecotopes with at least one infested site comprised 1118 (64%) inspected sites. Among them, storerooms had the highest prevalence of site infestation (19.1%; *n*, number of sites inspected = 136), followed by chicken coops (13.5%; *n* = 96), domiciles (12.1%; *n* = 256), chicken nesting structures (11.8%; *n* = 178), kitchens (11.3%; *n* = 106), and small granaries (8.0%; *n* = 25). Chicken nests (4.3%; *n* = 69) and goat and pig corrals (1.6%; *n* = 252) were less frequently infested (Fig. 3). All infested granaries and kitchens were colonized, followed by chicken coops or nests (not “nideros”) and domiciles (90–95%), storerooms (89%), and goat and pig corrals (75%). Chicken nests and domiciles had the lowest median bug abundance (1–2) whereas kitchens had the highest (15), followed by storerooms, chicken nesting structures, and chicken coops (6–7.5).

### Spatial patterns

**Fig. 4:**
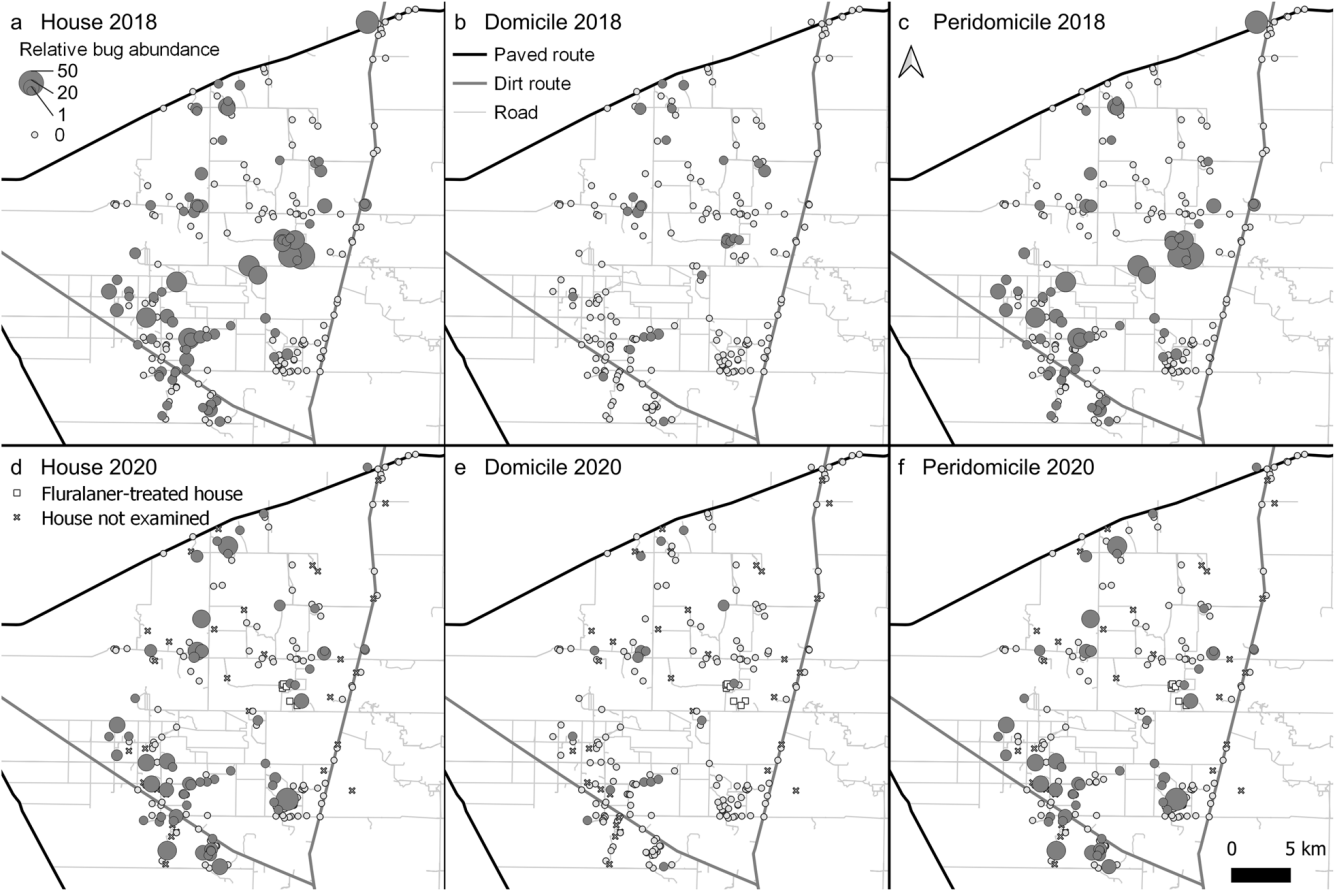
Spatial distribution of house (**a**–**d**), domestic (**b**–**e**), and peridomestic (**c**–**f**) abundance of *Triatoma infestans* as determined by TMC in Castelli in 2018 (**a**–**c**) and 2020 (**d**–**f**)

The spatial point patterns of infestation and bug abundance at the house, domicile, and peridomicile levels in 2018 and 2020 are shown in Fig. 4a–c and Fig. 4d–f, respectively. Baseline house and peridomestic infestations were widespread throughout the study area, while domestic infestations were detected in 7 of the 11 village groups (Fig. 4a–c). Broadly similar patterns were observed in 2020.

**Fig. 5:**
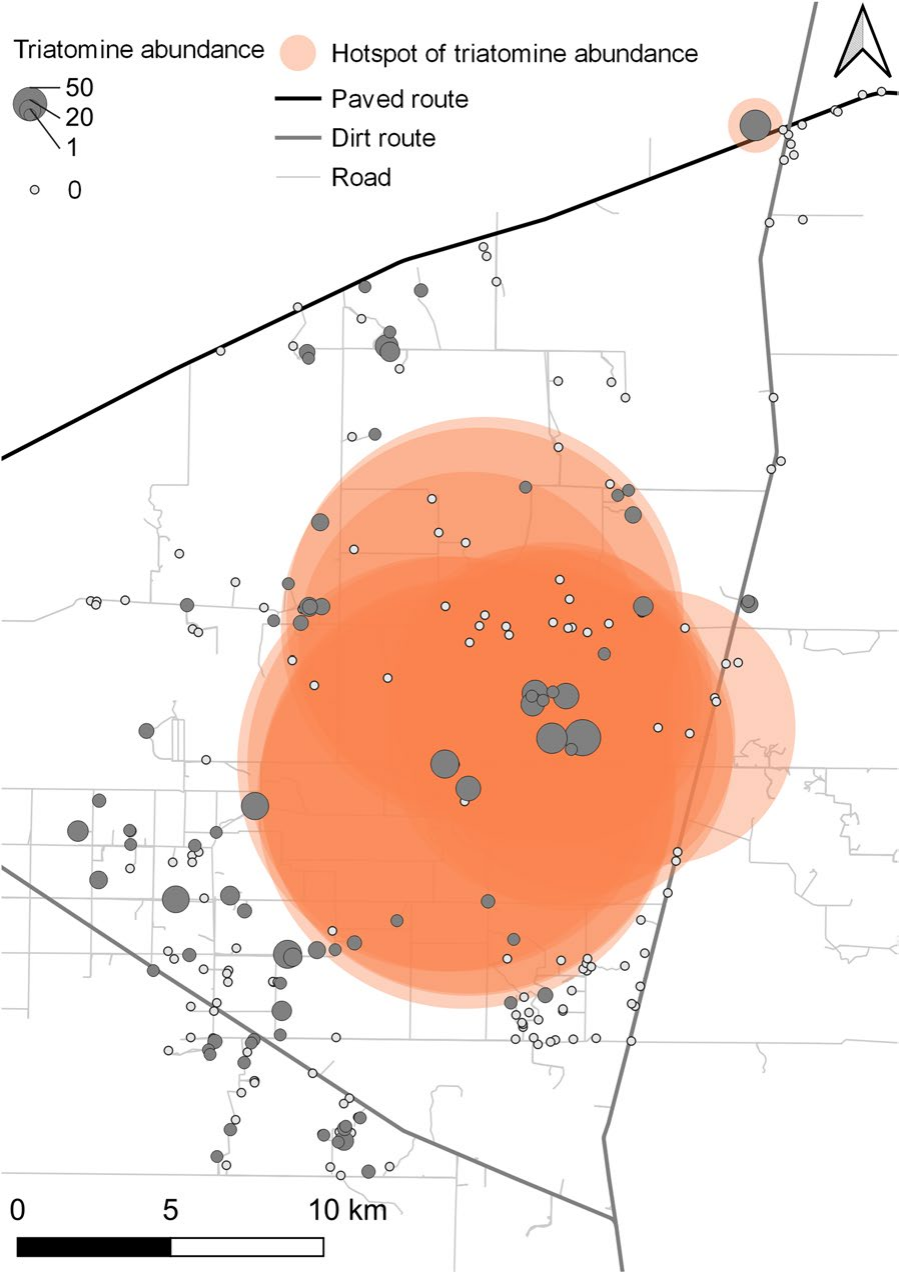
Spatial distribution of local hotspots for the relative abundance of *Triatoma infestans* per house as determined by TMC in Castelli at baseline, 2018

Baseline house infestation was significantly clustered on the global level between 540 and 1400 m (Fig. S1a). On average, for every infested house there was a higher probability of finding another infested house within this distance range than expected by chance. The relative abundance of *T. infestans* per house did not show global aggregation (weighted K-function) (Fig. S1b). Local spatial analysis identified significant clusters of high bug abundance within 100–6800 m (Gi*(d) > 3.71, *P* < 0.05) (Fig. 5). Of 234 study houses, 18 were members of the local clusters located in the central-eastern section of the study area, and only one house belonged to the northern cluster (El Cruce). These hotspots of bug abundance were located within 100–5400 m at El Asustado (including 4 houses), 800–4900 m and 6400–6500 m at El Ñandú (8 houses), 100–300 and 3300–8800 m at La Rinconada (5 houses), at 5600 m at El Juramento (1 house), and 100–900 m at El Cruce (1 house). All except El Cruce (which lacked data on RR_50_) had RR_50_ > 100 around 2010–2013 [57]. The median house bug abundance in village groups within the identified hotspots (range, 10–42) consistently exceeded the median values recorded in other villages (range, 1–11) (Table S1).

### Housing characteristics, sociodemographic variables, and house infestation

Most of the domiciles had > 1 bedroom (65.8%) and > 6 years (72%), displaying high refuge availability (62%), walls with many cracks (60.8%), brick-cement walls (79.6%), and metal sheet roofs (94.2%), and had > 5 domestic or peridomestic sites inspected for infestation (68%) at baseline (Table S2).

Among sociodemographic characteristics, 53.8% of households had < 1.7 residents per sleeping quarter, 74.7% had a goat-equivalent index of ≥ 20, and 93.1% did not keep poultry indoors (Table S3). Many households had > 20 chickens per house (42.9%) and ≥ 5 dogs (20.3%). According to householder reports, government-sponsored vector control personnel had sprayed insecticides in 44.2% of the study houses within the previous 3 years; the reported treatments were apparently selective as all but one comprised villages with past evidence of resistance. Most households reportedly applied domestic insecticides (79.7% of 231 houses) mainly in domiciles (74%; 136 of 184 houses). Among households reporting insecticide use, they mainly applied aerosol sprays (55.4%) containing carbamates, organophosphates, and pyrethroids, followed by acaricides (29%), pyrethroids applied in a powder or wet suspension though not always using backpack sprayers (19.9%), coils, or tablets [12.6%, carbamates (9.1%) and others (Table 2)]. Overall, 51% of households reported using only one type of insecticide; 32% used two, and 17% applied three or four types.

**Table 2:** Type of insecticides, acaricides, and repellents applied in households that reported using insecticides (*n* = 231) to fight nuisance pests in Castelli at baseline, 2018

Type of chemical product used in domiciles	No. of households reporting use (%)	
Aerosol insecticide sprays^a^	128	(55.4)
Concentrated phenolic disinfectant, chlorocresol^b^	67	(29.0)
Liquid pyrethroids (deltamethrin, cypermethrin)^c^	46	(19.9)
Mosquito coils, tablets^d^	29	(12.6)
Dust carbamates^e^	21	(9.1)
Unspecified	19	(8.2)

^a^Including pyrethrins and pyrethroids (i.e. cypermethrin). Additional active ingredients such as carbamates, organophosphates, and neonicotinoids were sometimes present

^b^Acaroin, also known as “Manchester fluid” and “creolin;” its main component is phenol

^c^In water suspension, e.g. K-Othrine R SC50 (Bayer)

^d^Containing a pyrethroid (i.e. transfluthrin)

^e^Commercially available as Sevin (Bayer)

Bivariate analyses of housing characteristics showed that age of the house since construction was significantly and negatively associated with baseline house infestation, and domestic area was positively associated with baseline house bug abundance (Table S2). The distance to the nearest infested house was negatively associated with both house infestation and bug abundance, with houses distant > 2000 m being at significantly lower risks. The remaining explanatory variables did not exhibit any significant association with either response variable.

Bivariate analyses of sociodemographic characteristics showed that the number of chickens and dogs per house and the goat-equivalent index were directly and significantly associated with baseline house infestation or bug abundance (Table S3). Additionally, the number of residents per house or per sleeping quarter were significantly and negatively associated with house-level bug abundance.

**Fig. 6:**
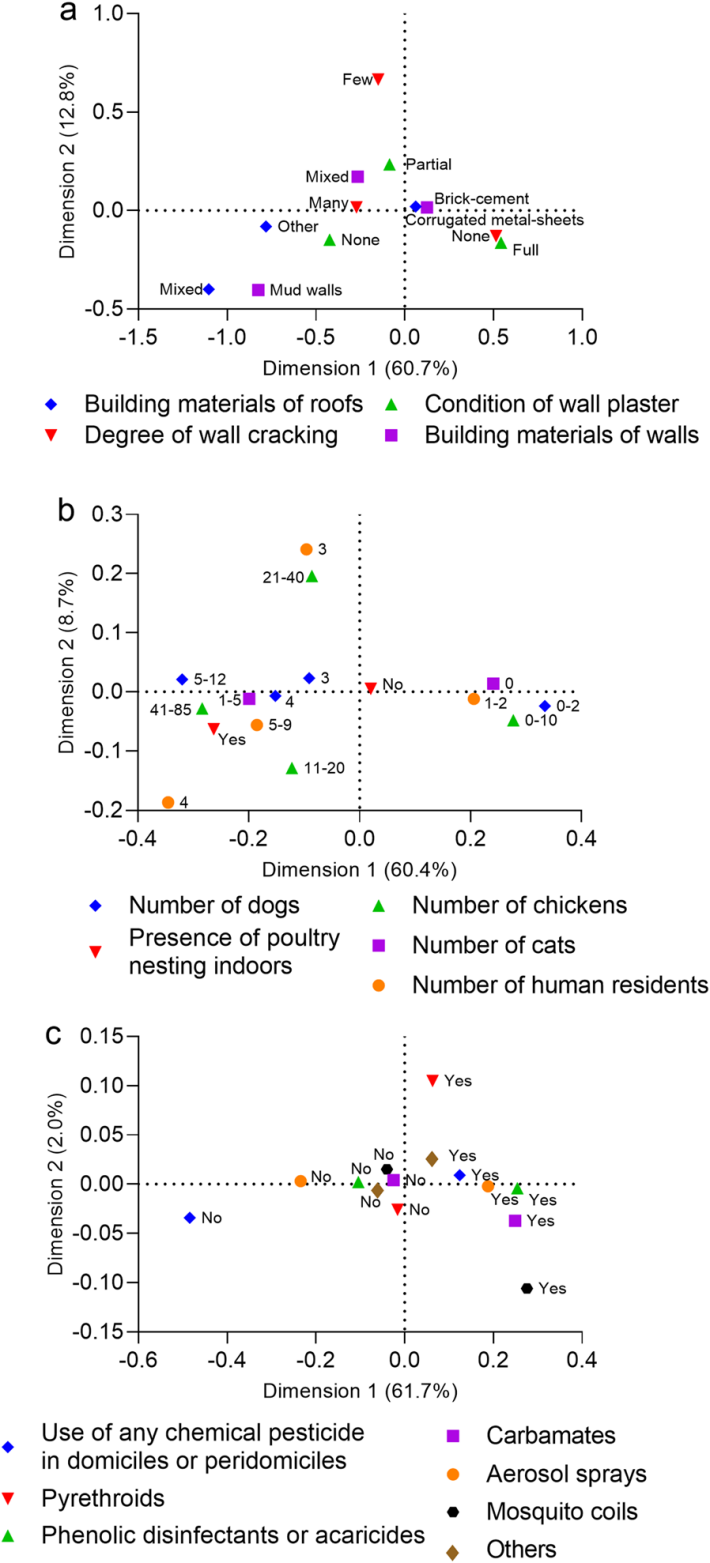
Biplots of the multiple correspondence analysis of building characteristics (**a**), domestic host availability (**b**), and household preventive practices (**c**) in Castelli at baseline, 2018

Using MCA, the amount of variation accounted for by dimension 1 in each biplot was 60.7% for building characteristics, 60.4% for domestic host availability, and 61.7% for household preventive practices (Fig. 6). In the biplot for building characteristics (Fig. 6a), the left side of the chart (showing negative index values) displayed categories representing low-quality buildings (e.g. ‘Other’ or ‘Mixed’ for roof materials, ‘Many’ for the degree of wall cracking, ‘No’ wall plaster, and ‘Mud walls’). Conversely, the categories indicating better quality buildings were on the right side of the chart and showed positive index values. As shown in Fig. 6b, more human residents were associated with owning more dogs, cats, chickens, and poultry indoors on the left of the plot, whereas categories with lower counts clustered on the right. Domestic insecticide use was most closely associated with the application of aerosol sprays, phenolic disinfectants, carbamates, and mosquito coils/tablets (Fig. 6c).

Householders raised livestock (i.e. cattle, pigs, and goats) in 189 (81.1%) of 233 households; 130 (68.8%) households reportedly treated their livestock with one or more antiparasitic products whereas 34 (18.0%) did not use any, and the remainder either did not know or respond. Among the 130 households that used antiparasitic products, 87.7% mainly applied pyrethroids (mostly cypermethrin) as the only pesticide for tick control or combined it with ivermectin; 6.2% used ivermectin or doramectin only, and 6.2% did not know the name of the product. The residual product left after treating livestock was used to treat the dogs (21.5%, mainly with cypermethrin). Livestock treatment with antiparasitic products was not associated with household pesticide use elsewhere within the house compound (*χ*^*2*^ = 3.09; *df* = 1; *P* = 0.08; *n* = 164). Among the 203 households responding whether they specifically treated the dogs with any product, 165 (81.3%) used at least one endoparasiticide or ectoparasiticide such as ivermectin, cypermethrin, and albendazole or praziquantel.

Multiple regression analysis showed that domestic infestation and bug abundance of *T. infestans* were significantly and negatively associated with indices for household preventive practices and housing quality, whereas domestic infestation was marginally associated with peridomestic bug abundance (Table 3). House-level infestation was negatively associated with household preventive practices and increased significantly with domestic host availability, whereas bug abundance was inversely related to the distance to the nearest infested house, household preventive practices, and housing quality (Table S4).

**Table 3:** Odds ratio (OR) and relative abundance (RA) for each predictor of domestic infestation (by logistic regression) and relative abundance of *T. infestans* (by negative binomial regression) for 209 domiciles in Castelli at baseline, 2018

	Domestic infestation		Domestic bug abundance	
Predictors	OR	(95% CI)	RA	(95% CI)
Distance to the nearest infested house (in m)	0.56	(0.29–1.10)	0.54	(0.18–1.66)
Household preventive practices index	0.59	(0.39–0.89)^a^	0.47	(0.29–0.78)^b^
Housing quality index	0.52	(0.35–0.77)^c^	0.38	(0.19–0.75)^b^
Domestic host availability index	0.97	(0.59–1.58)	1.12	(0.52–2.41)
Goat-equivalent index	0.10	(0.00–5.45)	0.20	(0.00–315.52)
Peridomestic bug abundance	1.38	(98–1.95)^d^	1.22	(0.62–2.40)

Logistic regression: Wald *χ*^*2*^ = 27.6, *df* = 6, *P* < 0.001; negative binomial regression: Wald *χ*^*2*^ = 21.5, *df* = 6, *P* = 0.002, 95% CI: 95% confidence interval

^a^0.01 < *P* < 0.05

^b^0.001 < *P* < 0.01

^c^*P* < 0.001

^d^*P* = 0.07

## Discussion

We found persistent house infestation in 2018 and 2020 at roughly similar levels as over 2005–2014 despite limited or marginal (2015–2020) control efforts, suggesting that the residual triatomine populations had slow recovery and propagation rates. As of 2018, all these villages (except El Cruce) harbored *T. infestans* populations with largely reduced mortality in discriminant dose assays for pyrethroid resistance (Gaspe et al. unpublished results). Our study disclosed a large spatial heterogeneity of house and domestic infestation and identified the location of several hotspots for targeted interventions. Such heterogeneous distributions were expected based on the high degree of genetic structuring of *T. infestans* populations at various scales, in part fueled by insecticide spraying [58], and the co-occurrence of resistant and susceptible triatomines among houses within the same village [28] or within the same house compound [59].

Despite limited or marginal insecticide pressure from 2015 onwards, the overall prevalences of house infestation in Castelli villages in 2018 (33.8%) and 2020 (31.6%) were virtually the same as the area-wide mean value estimated for the 2005–2014 period (33.7%) (Table 1). However, while the mean prevalence of domestic infestation decreased by 50% (from 24.1 to 12.2%) between the 2005 and 2014 period and 2018, mean peridomestic infestation increased by 62% (from 16.5 to 26.7%, respectively). For comparison, in rural villages of Santiago del Estero (dry Chaco) where *T. infestans* populations had no detectable pyrethroid resistance, house infestation and bug abundance recovered quickly to preintervention values within 4–7 years in the absence of control actions [4]. In Avia Terai (Chaco), a district embedded in a radically different agricultural landscape with sparse findings of moderate pyrethroid resistance, rural villages displayed a similar prevalence of house (42.4%) and domestic infestation (9.9%) (43) as in Castelli.

This retrospective comparison should be treated with caution in the light of several sources of variation between data sets created for different purposes. Significant data comparison issues include the design of a triatomine control campaign across a decade versus a longitudinal survey including supervised evaluations of site-level infestation combined with householder questionnaires, partial overlapping of the study villages and houses between data sets, village-wide versus house-based infestation data, and poorly defined search efforts for triatomines across a prolonged control campaign. Whether household preventive practices changed over time and major housing improvements set in also remain uncertain: nearly 30% of all study houses reportedly had < 6 years as of 2018. All assessments of house infestation by TMC (the reference method) underestimate true infestation when triatomines are at low density [60]; this is further compounded by the lower responsiveness of resistant triatomines to pyrethroid-based dislodging aerosols [61]. In a small comparison trial among TMCs, sticky traps, and householder notifications of *T. infestans* in Castelli villages, TMC outperformed other methods in peridomestic sites (detecting 82% of existing infestations) and missed several low-density domestic infestations detected by sticky traps [62]. Using the relative sensitivity of TMC in domestic sites (0.57) in [62] to approximately calibrate the observed domestic estimate of TMC (12.2%) in the current study yields an adjusted domestic infestation of 21.4% (12.2/0.57). These differences between observed and adjusted estimates do not modify our qualitative conclusions on domestic and peridomestic infestation levels between 2005–2014 and 2018. By contrast, the closed cohort of study houses inspected twice by TMC over 2018–2020 is free from those reservations; the slight difference in the timing of triatomine surveys during the hot season (mid-spring versus late summer) is unlikely to affect the performance of TMC. TMC-based estimates of house infestation or triatomine abundance may provide an internally valid measure of relative change as long as standardized procedures and bias remain approximately constant over time [21].

One plausible explanation of the slow recovery rates of house infestation and bug abundance points to the fitness costs of pyrethroid resistance, which affects the vital rates of *T. infestans*. In various experiments, pyrethroid resistance induced pleiotropic effects that prolonged the duration of late-stage nymphs and decreased female fecundity and fertility rates [40], mating frequency and egg hatching success [64], and walking dispersal of adult females [41]. When infected with *T. cruzi*, pyrethroid-resistant *T. infestans* exhibited ~ 60% greater bloodmeal size, lower defecation rates, and lower densities of metacyclic trypomastigotes than infected, pyrethroid-susceptible triatomines [63]. Using a stage-structured matrix model, experimental cohorts of *T. infestans* with high (Castelli) and moderate (Avia Terai) pyrethroid resistance were barely able to replace their numbers, while the susceptible (reference) cohort displayed a positive population growth rate close to other estimates in the literature [64]. This adds crucial support for the slow recovery rate of *T. infestans* populations in Castelli.

The spatial patterns of infestation and triatomine abundance were heterogeneous at the village and house levels. House-level bug abundance was significantly aggregated in the central-eastern section of the study area, including the four villages that had displayed maximum pyrethroid resistance approximately a decade before [31], thereby suggesting persisting infestation patterns over space-time. House infestation and triatomine abundance across Castelli villages were usually greater in peridomestic than domestic habitats, as in other rural locations of the Argentine Chaco with other characteristics [6, 9, 21, 65, 66]. Various structures used by chickens, including storerooms and kitchens, were key peridomestic ecotopes based on their large site infestation, colonization, and bug abundance levels [9, 42, 43]. This explains why an increasing triatomine abundance in peridomestic habitats increased the odds of domestic infestation with *T. infestans*, as recorded across the dry Chaco (e.g. [5, 6, 67] and in other triatomine species elsewhere [68, 69]. This connected dynamics fueled by the active and passive dispersal of triatomines in either direction is crucial for planning effective control strategies when the vector thrives in diverse habitats (e.g. [70]) and displays heterogeneous resistance levels.

Our study corroborates that the well-established risk factors for house infestation with susceptible *T. infestans* populations hold as well for the mixture of pyrethroid-resistant and susceptible triatomine populations infesting the study area. Housing quality and household preventive practices exerted negative effects on both domestic infestation and triatomine abundance across villages at baseline. Both findings are consistent with those obtained by multimodel analysis of preintervention domestic infestation in an area of Pampa del Indio with marginal pyrethroid resistance [15]. They are also in qualitative agreement with the outcomes of other risk-factor analyses of house infestation with *T. infestans* in Bolivia [10], Brazil [7], and elsewhere in northern Argentina [9, 14].

The housing quality index for domiciles synthesized the multiple dimensions of building characteristics, representing both the availability and quality of refuges for triatomines as determined by the types of wall and roof materials and condition of wall plaster [9, 14, 43]. The amount of physical space affects all vital rates and limits the abundance of *T. infestans* populations, as shown experimentally in closed chicken houses [8]. Housing quality may also reflect householders’ attitudes, means, skills, and labor for building maintenance and construction, as does the execution of preventive practices. Castelli domiciles mainly had brick-and-cement walls and corrugated metal roofs; these factors also explained the low domestic infestation levels recorded in Avia Terai [43]. Although housing improvements cannot fully prevent a domestic infestation because of the existence of other suitable refuges for triatomines, such as beds and household goods [9], they severely constrain bug population growth and may improve the effectiveness of insecticide applications. While housing improvement provides a set of health benefits beyond triatomine control, sustainable disease prevention in remote rural regions typically requires an integrated strategy including community involvement and vector management [71].

The index for household preventive practices based on reported pesticide usage was negatively associated with domestic infestation and bug abundance in Castelli despite the evidence of pyrethroid resistance. Other studies revealed similar effects of pesticide usage in areas with moderate or no pyrethroid resistance across the Argentine Chaco [9, 14, 15]. In Castelli, most households applied pesticides in domiciles and used low-concentration sprays primarily against mosquitoes and sandflies, with relatively fewer households using non-pyrethroid insecticides known to be effective for triatomines such as carbamates and ivermectin (see below). Pyrethroids have repellent and sublethal effects on triatomines, whereas the effects of acaricides or other substances are ill defined. This set of products and practices may explain the negative relationship between pesticide use and domestic infestation or bug abundance, although householder responses are typically affected by recall bias and could not be verified. Conversely, a substantial fraction of households consistently reported that official triatomine control personnel sprayed insecticide over 2015–2018, for which we found no official record. Despite the well-known limitations of questionnaire surveys and the lack of precision on usual application sites, doses, and frequency, our survey points to widespread pesticide use (mostly pyrethroids) partly associated with livestock raising and spillover use on domestic hosts and (peri)domestic premises.

The household use of insecticides was directly associated with the administration of various ectoparasiticides to livestock (mainly cypermethrin for ticks). The widespread occurrence and greater number of cattle in Castelli (63% of households owned cattle) contrasted with the patterns recorded across Pampa del Indio municipality, varying widely from 11% (Area 3) to 43% (Area 1) over 2008–2015. A large fraction of Castelli households frequently administered (mostly) ivermectin and cypermethrin, and posttreatment residues were applied to corrals and dogs. Ivermectin reduced the survival of *T. infestans* [72] depending on the mode of application [73] and goats treated with pour-on cypermethrin killed *T. infestans* over a month posttreatment depending on the dose [74]. Whether the combined use of pesticides in Castelli, partly associated with livestock raising, exerted negative impacts on (peri)domestic triatomine populations is a matter for further research.

The domestic host availability index explained variation in house infestation, though not in bug abundance, suggesting that the household number of humans and domestic animals apparently imposed little or no limitation to the low-density triatomine populations we encountered. This is consistent with previous results (reviewed in [4]) and the fact that the host-feeding success of *T. infestans* is maximal at low vector densities [75]. Nevertheless, bivariate analyses pinpointed significant and positive effects of chicken and dog numbers on one or both infestation metrics.

Our findings advocate for a multifaceted strategy to control pyrethroid-resistant *T. infestans* populations by integrating targeted triatomine surveillance with environmental management measures and judicious use of alternative insecticides with acceptable toxicity profiles, thereby leading to more sustainable and effective control efforts. Administration of endo-ectoparasiticides to domestic companion animals may provide a supplementary tool for controlling triatomines. Fluralaner administered to dogs reduced the relative abundance of *T. infestans* and its infection with *T. cruzi* in a small field trial in Castelli [76] and killed *Triatoma gerstaeckeri* after exposure to experimentally inoculated chickens [77]. Modification of habitats associated with persistent foci of *T. infestans* or *T. sordida* (e.g. structures occupied by chickens) offers another viable entry point for intervention with adaptive management practices tailored to specific scenarios. Improvement of domestic premises and goat corrals with appropriate technology minimized the availability of shelters and reduced triatomine infestation [78, 79]. Suppressing the triatomine populations with high pyrethroid resistance may help contain their regional spread by passive transportation. The recent introduction and establishment of *T. infestans* in Colima, Mexico [80], are reminders of the ongoing global process of biological invasions.

Several gaps in knowledge surfaced over the course of this research. One includes a lack of studies integrating the spatiotemporal dynamics of triatomine populations in pyrethroid-resistant areas and detection of pyrethroid resistance at meaningful operational scales from sets of contiguous villages up to the district level. While there are examples of modeling infestation and control dynamics over a decade long in Cochabamba and Bolivia [81] and of detailed house distribution of RR_50_ in a small village in Castelli [82], the challenge of addressing both processes simultaneously is still unmet. A second gap relates to the lack of understanding of the population-level impacts of pyrethroid resistance on triatomine infection and transmission of *T. cruzi* (see below). Third, searches for correlates of triatomine pyrethroid resistance have usually focused on the intensity of pyrethroid applications made by control programs, but other relevant sources associated with crop [83] and cattle protection deserve attention as they may operate in parallel though on a more continuous basis than sporadic, program-based triatomine control actions.

## Conclusions

*Triatoma infestans* populations in Castelli’s large focus of high pyrethroid resistance persisted at roughly similar values despite limited or marginal control actions, showing slow recovery rates and reduced capacity of propagation over a 5-year period. While this situation may not be as bleak as may have been predicted, a fraction of the houses remained under risk of vector-borne transmission [84], as indexed by domestic infestation or abundance and triatomine infection with *T. cruzi* [76]. Our findings have several implications for improved triatomine control in areas with pyrethroid resistance and beyond. First, while area- or village-specific pyrethroid resistance levels provide important information (indexed by RR_50_), they alone cannot be used to gauge the status of transmission risk at the relevant operational scales, nor can they predict house infestation dynamics. A similar argument has recently been made in the context of malaria control and pyrethroid-resistant mosquitoes [85]. In our study area, infestation indices frequently displayed large variation between and within villages in proximity; this means that only a fraction of houses in a hotspot or its proximities was infested. Second, the available evidence is insufficient to draw sweeping generalizations across the affected region where pyrethroid resistance emerged > 20 years ago: the long-term fate of *T. infestans* populations in the historical foci of pyrethroid resistance in northern Argentina and across Bolivia is yet to be disclosed. Understanding the spatiotemporal dynamics of the house infestation process in the context of heterogeneous pyrethroid resistance is crucial for developing and implementing effective control actions. The identification of spatial clusters provides valuable information for guiding control actions to prioritized areas, risk assessment, and resource allocation, whereas the hotspots of bug abundance point to areas with higher risks of vector-borne transmission. The issue of pyrethroid-resistant hotspots acquires another dimension in sparsely populated rural areas embedded in a mosaic of foci to which the spray teams have difficult access. A precise identification of such hotspots may assist in delivering more cost-effective interventions to block vector-bone transmission and suppress infestation and vector-borne transmission.

### Supplementary Information

Additional file 1: Figure S1 Global spatial analysis of house infestation (A) and abundance of *Triatoma infestans* per house (B) in Castelli, 2018. L(r): Linearized Ripley’s K-function where r is the distance (in m). The black line shows the expected distribution, the confidence envelope (in gray), while the red line indicates the observed pattern. The arrows indicate the range in which the observed L (d) exceeds the confidence envelope

Additional file 2: Table S1 Prevalence of house infestation, median relative abundance, and total catch of *Triatoma infestans* at the house, domicile, and peridomicile levels by village in Castelli at baseline, 2018

Additional file 3: Table S2 Prevalence of house infestation and relative abundance of *Triatoma infestans* according to housing characteristics in Castelli at baseline, 2018. Continuous and discrete variables were categorized according to their quartiles.

Additional file 4: Table S3 Prevalence of house infestation and relative abundance of *Triatoma infestans* according to sociodemographic variables in Castelli at baseline, 2018. Continuous and discrete variables were categorized according to their quartiles.

Additional file 5: Table S4 Odds ratio (OR) and relative abundance (RA) for each variable regarding house infestation (logistic regression) and relative abundance of *Triatoma infestans* (negative binomial regression) for a model including 219 inhabited houses in Castelli at baseline, 2018. For logistic regression: Wald *χ²* = 29.4, *df* = 5, *P* < 0.001; for negative binomial regression: Wald *χ²* = 51.3, *d.f*. = 5, *P* < 0.001.

Additional file 6: Table S5 Database used for analysis.

## Data Availability

All relevant data are provided in Additional file 6: Table S5.

## Abbreviations

TMC: Timed manual collections
RR_50_: Ratio between the median lethal dose (LD50) of a tested field population and the LD50 of the susceptible or reference population
MCA: Multiple correspondence analysis
