# Supplementary figures and images for: Slow recovery rates and spatial aggregation of Triatoma infestans populations in an area with high pyrethroid resistance in the Argentine Chaco
Source: Parasit Vectors. 2024 Jul 2;17:287. doi: 10.1186/s13071-024-06366-7 (PMC11220979; doi:10.1186/s13071-024-06366-7)

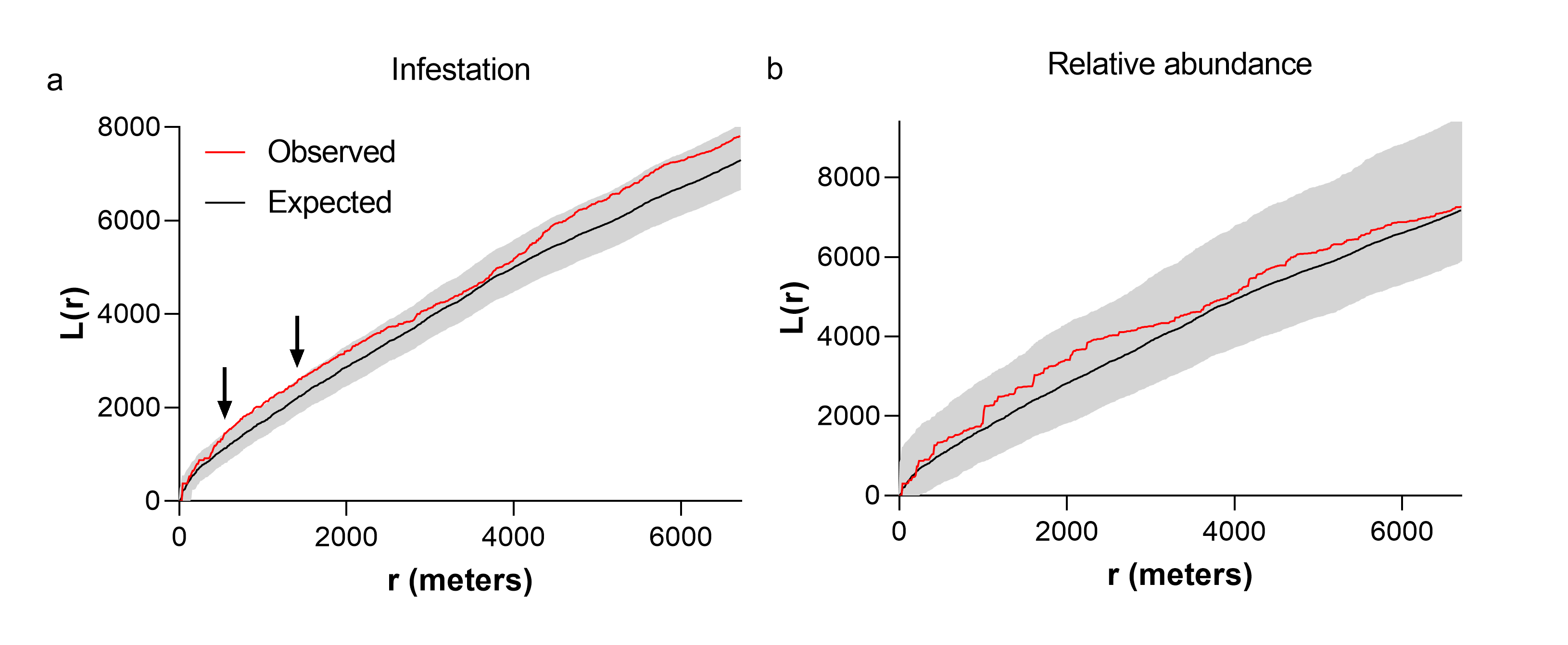

Supplement: Supplementary file 1 — Additional file 1: Figure S1 Global spatial analysis of house infestation (A) and abundance of Triatoma infestans per house (B) in Castelli, 2018. L(r): Linearized Ripley’s K-function where r is the distance (in m). The black line shows the expected distribution, the confidence envelope (in gray), while the red line indicates the observed pattern. The arrows indicate the range in which the observed L (d) exceeds the confidence envelope [file 13071_2024_6366_MOESM1_ESM.tif]
