# Supplementary material for: Slow recovery rates and spatial aggregation of Triatoma infestans populations in an area with high pyrethroid resistance in the Argentine Chaco
Source: Parasit Vectors. 2024 Jul 2;17:287. doi: 10.1186/s13071-024-06366-7 (PMC11220979; doi:10.1186/s13071-024-06366-7)
Supplement: Supplementary file 2 — Additional file 2: Table S1 Prevalence of house infestation, median relative abundance, and total catch of Triatoma infestans at the house, domicile, and peridomicile levels by village in Castelli at baseline, 2018 [file 13071_2024_6366_MOESM2_ESM.docx]

**Table S1** Prevalence of house infestation, median relative abundance and total catch of *Triatoma infestans* at the house, domicile, and peridomicile levels by village in Castelli at baseline, 2018

| Villages^a^ | Prevalence of infestation  (No. inspected) | | | Median bug abundance per infested habitat  (1^st^-3^rd^ quartiles, total catch) | | | |  |
| --- | --- | --- | --- | --- | --- | --- | --- | --- |
|  | House | Domicile | Peridomicile | House | Domicile | Peridomicile | | |
| La Maravilla, San Agustín, and La Anta | 46.2 (26) | 30.8 (26) | 23.1 (26) | 5 (3.5-11.5, 95) | 5 (2-9.5, 46) | | 3.5 (2-15, 49) | |
| El Asustado | 29.4 (17) | 0 (15) | 29.4 (17) | 13 (7-41, 124) | - | | 13 (7-41, 124) | |
| El Malhá and Las Flores | 20.0 (10) | 0 (10) | 20.0 (10) | 9 (8-10, 18) | - | | 9 (8-10, 18) | |
| La Rinconada | 33.3 (6) | 16.7 (6) | 33.3 (6) | 31 (27-35, 62) | 3 (-, 3) | | 29.5 (24-35, 59) | |
| La Unión | 58.8 (17) | 6.7 (15) | 52.9 (17) | 7.5 (1-19, 119) | 1 (-, 1) | | 8 (3-19, 118) | |
| La Gerónima | 11.1 (36) | 0 (36) | 11.4 (35) | 7.5 (5.5-9, 29) | - | | 7.5 (5.5-9, 29) | |
| Campo Florido | 39.5 (38) | 6.3 (32) | 36.8 (38) | 5 (2-8, 82) | 3 (1-5, 6) | | 5 (2-7, 76) | |
| El Cruce | 6.7 (15) | 0 (15) | 6.7 (15) | 42.0 (-, 42 ) | - | | 42 (-, 42) | |
| El Juramento | 41.2 (17) | 31.3 (16) | 18.8 (16) | 10 (4-13, 66) | 4 (1-10, 26) | | 13 (12-15, 40) | |
| El Ñandú | 38.1 (21) | 30.0 (20) | 28.6 (21) | 11.5 (5-26.5, 119) | 5.5 (4-9, 35) | | 16 (3-20, 84) | |
| La Esperanza | 41.9 (31) | 12.9 (31) | 32.3 (31) | 9 (1-15, 132) | 2 (1-6, 14) | | 9.5 (6-16, 118) | |
| Overall | 33.8 (234) | 12.2 (222) | 26.7 (232) | 8 (3-15, 888) | 4 (1-9, 131) | | 8 (3-16, 757) |  |
