# Supplementary material for: Slow recovery rates and spatial aggregation of Triatoma infestans populations in an area with high pyrethroid resistance in the Argentine Chaco
Source: Parasit Vectors. 2024 Jul 2;17:287. doi: 10.1186/s13071-024-06366-7 (PMC11220979; doi:10.1186/s13071-024-06366-7)
Supplement: Supplementary file 3 — Additional file 3: Table S2 Prevalence of house infestation and relative abundance of Triatoma infestans according to housing characteristics in Castelli at baseline, 2018. Continuous and discrete variables were categorized according to their quartiles. [file 13071_2024_6366_MOESM3_ESM.docx]

**Table S2** Prevalence of house infestation and relative abundance of *T. infestans* according to housing characteristics in Castelli at baseline, 2018. Continuous and discrete variables were categorized according to their quartiles.

| Variables | No. of inspected houses (%) | Prevalence of house infestation ^a^ | OR (95% CI)† | Median bug abundance (1st-3rd quartiles) ^b^ | IRR (95% CI) |
| --- | --- | --- | --- | --- | --- |
| Building materials of walls | | | | | |
| Brick-cement | 176 (79.6) | 31.3 | 1 | 8 (4-16) | 1.0 |
| Mixed | 27 (12.2) | 48.1 | 2.0 (0.9-4.7) | 6 (3-12) | 1.5 (0.5-4.9) |
| Mud | 18 (8.1) | 27.8 | 0.9 (0.3-2.5) | 4 (1-10) | 0.4 (0.1-1.7) |
| Total | 221 |  |  |  |  |
| Degree of wall cracking | | | | | |
| None | 74 (34.1) | 28.4 | 1 | 10 (5-17) | 1.0 |
| Few | 11 (5.1) | 27.3 | 1.0 (0.2-4.0) | 12 (3-30) | 1.0 (0.2-6.1) |
| Many | 132 (60.8) | 35.6 | 1.4 (0.8-2.6) | 7 (3-10) | 0.7 (0.3-1.7) |
| Total | 217 |  |  |  |  |
| Condition of wall plaster | | | | | |
| Full | 66 (30.4) | 27.3 | 1 | 11 (6-19) | 1.0 |
| Partial | 85 (39.2) | 35.3 | 1.5 (0.7-3.0) | 8 (3-12) | 0.7 (0.3-1.8) |
| None | 66 (30.4) | 34.8 | 1.4 (0.7-3.0) | 6 (3-11) | 0.7 (0.2-1.8) |
| Total | 217 |  |  |  |  |
| Building materials of roofs | | | | | |
| Corrugated metal sheets | 210 (94.2) | 32.4 | 1 | 8 (4-15) | 1.0 |
| Other | 13 (5.8) | 46.2 | 1.8 (0.6-5.6) | 3 (1-5) | 0.5 (0.1-2.5) |
| Total | 223 |  |  |  |  |
| Refuge availability in domicile ^c^ | | | | | |
| 3 | 81 (38.0) | 25.9 | 1 | 10 (7-17) | 1.0 |
| 4 | 86 (40.0) | 34.9 | 1.5 (0.8-3.0) | 6.5 (1-9) | 0.85 (0.3-2.1) |
| 5 | 46 (21.6) | 39.1 | 1.8 (0.8-4.0) | 7.5 (4-12) | 1.1 (0.4-3.1) |
| Total | 213 |  |  |  |  |
| Age of house (years) | | | | | |
| 0.3-6 | 49 (28.5) | 30.6 | 1 | 10 (4-16) | 1.0 |
| 6.5-11 | 39 (22.7) | 12.8 | 0.3 (0.1-1.0)* | 15 (13-16) | 0.7 (0.2-2.4) |
| 12-20 | 46 (26.7) | 41.3 | 1.6 (0.7-3.7) | 6 (2-9) | 1.0 (0.3-3.4) |
| 21-50 | 38 (22.1) | 34.2 | 1.2 (0.5-2.9) | 8 (4-10) | 0.8 (0.2-2.8) |
| Total | 172 |  |  |  |  |
| No. of bedrooms | | | | | |
| 1 | 77 (34.2) | 29.9 | 1 | 7 (3-10) | 1.0 |
| 2 | 78 (34.7) | 35.9 | 1.3 (0.7-2.6) | 8 (4-16.5) | 1.5 (0.6-3.8) |
| 3 | 52 (23.1) | 28.8 | 1.0 (0.4-2.1) | 7 (4-13) | 1.1 (0.4-3.0) |
| 4-6 | 18 (8.0) | 44.4 | 1.9 (0.6-5.4) | 11 (6-21) | 2.0 (0.5-9.0) |
| Total | 225 |  |  |  |  |
| Domestic area (m^2^) | | | | | |
| 5-24 | 54 (27.6) | 24.1 | 1 | 5 (1-9) | 1.0 |
| 25-42 | 48 (24.5) | 35.4 | 1.7 (0.7-4.1) | 7 (1-15) | 2.8 (0.9-9.3) |
| 43-75 | 45 (23.0) | 22.2 | 0.9 (0.4-2.3) | 5 (3-8) | 0.9 (0.3-3.2) |
| 76-100 | 49 (25.0) | 32.7 | 1.5 (0.6-3.7) | 16.5 (7.5-28.5) | 4.7 (1.4-15.2)* |
| Total | 196 |  |  |  |  |
| No. of potentially suitable sites for triatomines | | | | | |
| 1-5 | 75 (32.1) | 34.7 | 1 | 6 (1-13) | 1.0 |
| 6-7 | 63 (26.9) | 28.6 | 0.8 (0.4-1.6) | 8 (4-23) | 1.4 (0.5-3.7) |
| 8-9 | 40 (17.1) | 30.0 | 0.8 (0.4-1.9) | 11 (6-16.5) | 1.2 (0.4-3.7) |
| 10-23 | 56 (23.9) | 41.1 | 1.3 (0.6-2.7) | 7 (3-10) | 1.4 (0.5-3.7) |
| Total | 234 |  |  |  |  |
| Distance to the nearest infested house (m) | | | | | |
| 22-486 | 59 (25.2) | 50.8 | 1 | 8 (3-16) | 1.0 |
| 487-1,071 | 58 (24.8) | 39.7 | 0.6 (0.3-1.3) | 7 (1-12) | 0.7 (0.3-2.0) |
| 1,072-2,023 | 59 (25.2) | 32.2 | 0.5 (0.2-1.0)* | 8 (6-19) | 0.7 (0.3-2.0) |
| 2,024-12,496 | 58 (24.8) | 12.1 | 0.1 (0.05-0.4)* | 10 (4-17) | 0.3 (0.1-0.9) * |
| Total | 234 |  |  |  |  |

a. House infestation was determined by the finding of at least one live bug using TMC.

b. Median bug abundance was calculated from the total number of live bugs collected by TMC in infested houses.

c. Domiciles with minimal refuge availability for triatomines (category 1 and 2) were absent.

† Includes inhabited houses.

OR: Crude odds ratio. RA: Relative abundance. 95% CI: 95% confidence interval. Households with missing data were excluded from each variable. IRR labeled in Stata output as 'incidence-rate ratios’, and their CIs were calculated from the estimated coefficients (b) of the negative binomial regression.

* *P* **≤** 0.05
