# Supplementary material for: Slow recovery rates and spatial aggregation of Triatoma infestans populations in an area with high pyrethroid resistance in the Argentine Chaco
Source: Parasit Vectors. 2024 Jul 2;17:287. doi: 10.1186/s13071-024-06366-7 (PMC11220979; doi:10.1186/s13071-024-06366-7)
Supplement: Supplementary file 4 — Additional file 4: Table S3 Prevalence of house infestation and relative abundance of Triatoma infestans according to sociodemographic variables in Castelli at baseline, 2018. Continuous and discrete variables were categorized according to their quartiles. [file 13071_2024_6366_MOESM4_ESM.docx]

**Table S3** Prevalence of house infestation and relative abundance of *T. infestans* according to sociodemographic variables in Castelli at baseline, 2018. Continuous and discrete variables were categorized according to their quartiles.

| Variables | No. of inspected houses (%) | Prevalence of house infestation^a^ | OR (95% CI)† | Median bug abundance (1st-3rd quartiles)^b^ | IRR (95% CI) |
| --- | --- | --- | --- | --- | --- |
| No. of human residents^c^ | | | | | |
| 1-2 | 113 (48.3) | 34.5 | 1 | 7 (4-16) | 1.0 |
| 3 | 40 (17.1) | 37.5 | 1.1 (0.5-2.4) | 10 (4-17) | 1.3 (0.5-3.6) |
| 4 | 27 (11.5) | 40.7 | 1.3 (0.5-3.1) | 9 (4-19) | 1.4 (0.4-4.5) |
| 5-9 | 54 (23.1) | 25.9 | 0.7 (0.3-1.4) | 4 (1-10) | 0.4 (0.2-1.0)* |
| Total | 234 |  |  |  |  |
| Overcrowding index^d^ | | | | | |
| 0.2-1.1 | 97 (43.1) | 39.2 | 1 | 9 (6-16) | 1.0 |
| 1.2-1.6 | 24 (10.7) | 33.3 | 0.8 (0.3-2.0) | 11 (3-29) | 1.0 (0.3-3.7) |
| 1.7-2.1 | 53 (23.6) | 28.3 | 0.6 (0.3-1.3) | 7 (1-9) | 0.4 (0.1-1.0)* |
| 2.2-6.0 | 51 (22.7) | 25.5 | 0.5 (0.2-1.1) | 8 (3-11) | 0.4 (0.2-1.1) |
| Total | 225 |  |  |  |  |
| Poultry indoors^e^ | | | | | |
| No | 215 (93.1) | 32.6 | 1 | 8 (4-16) | 1.0 |
| Yes | 16 (6.9) | 43.8 | 1.6 (0.6-4.5) | 7 (3-8) | 0.7 (0.2-3.2) |
| Total | 231 |  |  |  |  |
| Goat-equivalent index^f^ | | | | | |
| 0-19 | 59 (25.3) | 30.5 | 1 | 5 (1-10) | 1.0 |
| 20-141 | 58 (24.9) | 36.2 | 1.3 (0.6-2.8) | 9 (6-23) | 2.7 (1.0-7.6)# |
| 142-326 | 58 (24.9) | 29.3 | 0.9 (0.4-2.1) | 12 (5-16) | 2.0 (0.7-5.7) |
| 327-9,276 | 58 (24.9) | 37.9 | 1.4 (0.6-3.0) | 7 (3-10) | 1.8 (0.6-5.0) |
| Total | 233 |  |  |  |  |
| No. of chickens | | | | | |
| 0-10 | 80 (34.3) | 23.8 | 1 | 6 (1-10) | 1.0 |
| 11-20 | 53 (22.8) | 26.4 | 1.2 (0.5-2.6) | 13.5 (7-16) | 2.0 (0.7-5.1) |
| 21-40 | 60 (25.8) | 38.3 | 2.0 (1.0-4.2)# | 7 (3-14) | 2.4 (1.0-6.2)# |
| 41-100 | 40 (17.2) | 57.5 | 4.3 (1.8-10.3)* | 8 (3-13) | 4.2 (1.5-12.0)* |
| Total | 233 |  |  |  |  |
| No. of dogs | | | | | |
| 0-2 | 82 (35.3) | 29.3 | 1 | 6.5 (1.5-16.5) | 1.0 |
| 3 | 56 (24.1) | 33.9 | 1.2 (0.6-2.6) | 8 (4-11) | 1.0 (0.4-2.6) |
| 4 | 47 (20.3) | 28.8 | 1.0 (0.5-2.3) | 6.5 (3-16) | 1.0 (0.4-2.9) |
| 5-12 | 47 (20.3) | 46.8 | 2.1 (1.0-4.5)* | 8 (4-17) | 1.7 (0.6-4.7) |
| Total | 232 |  |  |  |  |
| No. of cats | | | | | |
| No | 105 (45.3) | 35.2 | 1 | 8 (4-15) | 1.0 |
| Yes | 127 (54.7) | 33.1 | 0.9 (0.5-1.6) | 8 (3-14) | 1.1 (0.5-2.3) |
| Total | 232 |  |  |  |  |
| Time since last reported insecticide spraying (years) | | | | | |
| 1-2 | 51 (26.8) | 25.5 | 1 | 5 (3-7) | 1 |
| 3 | 33 (17.4) | 39.4 | 1.9 (0.7-4.9) | 5 (1-8) | 3.0 (0.8-11.1) |
| 4-5 | 28 (14.7) | 32.1 | 1.4 (0.5-3.8) | 7 (6-9) | 2.5 (0.6-10.0) |
| >6, never or not recalled | 78 (41.1) | 29.5 | 1.2 (0.5-2.7) | 13 (3-17) | 2.9 (1.0-8.4)* |
| Total | 190 |  |  |  |  |
| Insecticide use^g^ | | | | | |
| No | 47 (20.3) | 42.6 | 1 | 10 (3.5-16.5) | 1.0 |
| Yes | 184 (79.7) | 32.1 | 0.6 (0.3-1.2) | 8 (3-14) | 0.7 (0.3-1.7) |
| Total | 231 |  |  |  |  |
| Household educational level^h^ | | | | | |
| 0-3.0 | 51 (26.7) | 39.2 | 1 | 7.5 (2.5-10) | 1.0 |
| 3.1-5.0 | 57 (29.8) | 28.1 | 0.6 (0.3-1.4) | 8 (5.5-14) | 1.1 (0.4-3.4) |
| 5.1-7.0 | 64 (33.5) | 28.1 | 0.6 (0.3-1.3) | 8 (1-16) | 0.9 (0.3-2.8) |
| 7.1-12.0 | 19 (10.0) | 15.8 | 0.3 (0.1-1.2) | 3 (1-8) | 0.2 (0.0-1.1) |
| Total | 191 |  |  |  |  |

a. House infestation was determined by the finding of at least one live bug using TMC.

b. Median bug abundance was calculated from the total number of live bugs collected by TMC in infested houses.

c. Number of human residents per house.

d. Number of human residents per sleeping quarter.

e. Nesting indoors.

f. Number of livestock (cows, pigs, goats) and poultry owned by the household in terms of goat biomass.

g. Domestic insecticide use refers to householder applications in domiciles or/and peridomiciles of at least one of the chemical products listed in Suppl. Table 3.

h. Household educational level was defined as the mean number of schooling years attained by household members aged 15 years old or more.

† Includes inhabited houses.

OR: Crude odds ratio. RA: Relative abundance. 95% CI: 95% confidence interval. Households with missing data were excluded from each variable. IRR, labeled in Stata output as 'incidence-rate ratios’, and their CIs were calculated from the estimated coefficients (b) of the negative binomial regression.

* *P* **≤** 0.05; # *P* = 0.06.
