# Supplementary material for: Slow recovery rates and spatial aggregation of Triatoma infestans populations in an area with high pyrethroid resistance in the Argentine Chaco
Source: Parasit Vectors. 2024 Jul 2;17:287. doi: 10.1186/s13071-024-06366-7 (PMC11220979; doi:10.1186/s13071-024-06366-7)
Supplement: Supplementary file 5 — Additional file 5: Table S4 Odds ratio (OR) and relative abundance (RA) for each variable regarding house infestation (logistic regression) and relative abundance of Triatoma infestans (negative binomial regression) for a model including 219 inhabited houses in Castelli at baseline, 2018. For logistic regression: Wald χ² = 29.4, df = 5, P < 0.001; for negative binomial regression: Wald χ² = 51.3, d.f. = 5, P < 0.001. [file 13071_2024_6366_MOESM5_ESM.docx]

**Table S4** Odds ratio (OR) and relative abundance (RA) for each variable regarding house infestation (logistic regression) and relative abundance of *T. infestans* (negative binomial regression) for a model including 219 inhabited houses in Castelli at baseline, 2018. For logistic regression: Wald *χ²* = 29.4, df = 5, *P* < 0.001; for negative binomial regression: Wald *χ²* =14.15, df = 5, *P* = 0.015

| Predictors | House infestation | | House bug abundance | |  |
| --- | --- | --- | --- | --- | --- |
|  | OR | (95% CI) | RA | (95% CI) | |
| Distance to the nearest infested house (in m) | 0.23 | (0.03-1.63) | 0.46 | (0.10-2.13) | |
| Household preventive practices index | 0.29 | (0.15-0.56)*** | 0.48 | (0.22-1.08) | |
| Housing quality index | 0.54 | (0.24-1.21) | 0.49 | (0.22-1.11) | |
| Domestic host availability index | 2.33 | (1.05-5.15)* | 1.24 | (0.44-3.47) | |
| Goat-equivalent index | 0.34 | (0.01-10.80) | 0.41 | (0.02-6.78) | |

CI: confidence interval.

* 0.01 < *P* < 0.05. ** 0.001 < *P* < 0.01. *** *P* < 0.001
